# Real Corn Bread

**Published:** 1859-01

**Authors:** 


### REAL CORN BREAD.

A corn dodger is not now what it used to be. Originally it was a corn meal dumpling. In very early Kentucky times, the universal dinner, winter and spring at every farm house is the state, was a piece of middling bacon, boiled with cabbage, turnips, greens, collards, or sprouts, cabbage sprouts, according to the season. The pot, if the family was a large one, contained about ten gallons, and was nearly filled with clean pure water, the middlings and the greens were put in at the proper time, to give them a sufficient cooking. Almost always the cook would make with water and corn meal and a little salt, dough balls, throw them into the pot, and boil them thoroughly with the rest. These were called *dodgers*, from the motion given them by the boiling water in the pot. They eat very well, and give a considerable variety to a dinner of bacon and collards. A dodger in modern times is corn bread baked in a roll about the size of your hand, and about three time as thick, and in my judgment is not a veritable first rate dodger, unless when on the table it bears the impress of the cook's fingers on it, in placing it in the oven to bake.

A pone of bread is corn bread baked in a skillet or small oven. The skillet or oven when at the proper heat is filled with corn dough, and baked, and when baked and turned out, is a pone of bread.

A hoe cake is not now what it used to be. I do not believe there will ever be any more good hoe cakes baked. I have an unextinguishable longing for hoe cake—real hoe cake, such a the black woman Jinny, my mother's cook always baked. It gets its name from the mode of baking. It was originally baked upon a hoe. An old hoe, (a hoe was one of our primitive implements of agriculture, but now almost out of use,) which had been worn bright, and the handle out, was placed upon live coals of fire, with the eye down, and on it the cake was baked. Now, hoe cake is baked upon a griddle, or was before cooking stoves came into use. Do you know what a griddle is? Of course you do. It just occurs to me, may not the cooking stove militate against hoe cake? The griddle I believe has been displaced by it altogether, and I now have an idea that good hoe cakes can be baked only on a hoe or on a grid-dle.

Corn dodger, corn pone and hoe cakes are different only in the baking. The meal is prepared for each, precisely in the same way. Take as much meal as you want, some salt, and enough pure water to knead the mass. Mix it well, let it stand some 15 or 20 minutes, not longer, as this will be long enough to saturate perfectly every particle of meal, bake on the griddle for hoe cake, and in the oven or skillett for dodger and pone. The griddle or oven must be made hot enough to bake, but not *to burn*, but with a quick heat. The lid must be heated also before putting it on the skillet or oven, and that heat must be kept up with coals of fire placed on it, as these must be around and under the oven. The griddle must be well supplied with live coals under it. The hoe cake must be put on thin, not more than or quite as thick as your fore finger; when brown, it must be turned, and both sides baked to a rich brown color. There must be no burning—baking is the idea. Yet the baking must be done with a quick lively heat, the quicker the better. Saleratus and soda, *procul o procul!* Let there be nothing but water and salt. G. W. W.

The above was written by a son of Kentucky, himself one of her best ornaments, and is authentic.

